# Renoprotective potentials of small molecule natural products targeting mitochondrial dysfunction

**DOI:** 10.3389/fphar.2022.925993

**Published:** 2022-07-15

**Authors:** Md. Ataur Rahman, Sumaya Akter, Debra Dorotea, Arpita Mazumder, Md. Naim Uddin, Md. Abdul Hannan, Muhammad Jahangir Hossen, Md. Selim Ahmed, Woojin Kim, Bonglee Kim, Md Jamal Uddin

**Affiliations:** ^1^ ABEx Bio-Research Center, Dhaka, Bangladesh; ^2^ Department of Pathology, College of Korean Medicine, Kyung Hee University, Hoegidong Dongdaemungu, Seoul, South Korea; ^3^ Korean Medicine-Based Drug Repositioning Cancer Research Center, College of Korean Medicine, Kyung Hee University, Seoul, South Korea; ^4^ Graduate School of Pharmaceutical Sciences, College of Pharmacy, Ewha Womans University, Seoul, South Korea; ^5^ Department of Biochemistry and Molecular Biology, Bangladesh Agricultural University, Mymensingh, Bangladesh; ^6^ Department of Animal Science, Patuakhali Science and Technology University, Dumki, Patuakhali, Bangladesh; ^7^ Department of Medicine, Surgery and Obstetrics, Faculty of Animal Science and Veterinary Medicine, Patuakhali Science and Technology University, Barisal, Bangladesh; ^8^ Department of Physiology, College of Korean Medicine, Kyung Hee University, Seoul, South Korea

**Keywords:** kidney diseases, mitochondrial dysfunction, small molecule natural products, traditional medicine, renoprotective effect

## Abstract

Kidney diseases, including acute kidney injury (AKI) and chronic kidney disease (CKD), have become critical clinical, socioeconomic, and public health concerns worldwide. The kidney requires a lot of energy, and mitochondria act as the central organelle for the proper functioning of the kidney. Mitochondrial dysfunction has been associated with the pathogenesis of AKI and CKD. Natural products and their structural analogs have been sought as an alternative therapeutic strategy despite the challenges in drug discovery. Many studies have shown that small-molecule natural products can improve renal function and ameliorate kidney disease progression. This review summarizes the nephroprotective effects of small-molecule natural products, such as berberine, betulinic acid, celastrol, curcumin, salidroside, polydatin, and resveratrol. Treatment with small-molecule natural products was shown to attenuate renal oxidative stress and mitochondrial DNA (mtDNA) damage and restore mitochondrial biogenesis and dynamics in the kidneys against various injury stimuli. Therefore, small-molecule natural products should be recognized as multi-target therapeutics and promising drugs to prevent kidney diseases, particularly those with mitochondrial dysfunction.

## Introduction

Kidney diseases are a major escalating public health issue globally associated with serious clinical complications (Liu et al., 2019; Li Q. et al., 2021; Wang D.-W. et al., 2021). Kidney injury is broadly classified into acute kidney injury (AKI) and chronic kidney disease (CKD). AKI is characterized by the rapid loss of renal excretory function within a short duration (hours to days) (Wang D.-W. et al., 2021). The common etiologies of AKI include ischemia, obstructive nephropathy (ON), nephrotoxins, and sepsis (Martínez-Klimova et al., 2020; Wang D.-W. et al., 2021). AKI has been recognized as a crucial risk factor for the occurrence and progression of CKD (Jiang et al., 2020), which involves the gradual loss of kidney function with reduced glomerular filtration rate and enhanced urinary albumin excretion, resulting in end-stage renal disease (ESRD) (Liu et al., 2019; Li Q. et al., 2021). Despite the availability of advanced supportive management and diagnosis, AKI and CKD have high morbidity and mortality due to less effective therapies (Gao et al., 2020; Li Q. et al., 2021; Wang D.-W. et al., 2021). Therefore, it is imperative to explore more efficacious therapeutic strategies to treat and prevent kidney disease progression (Li Q. et al., 2021; Wang D.-W. et al., 2021).

Natural products and their structural analogs have made a major contribution to pharmacotherapy despite the challenges in drug discovery. Consumers are using naturally-derived substances in the form of herbal medications or nutraceuticals to avoid the potential adverse effects of the pharmaceutical drugs (Dias et al., 2012). According to the World Health Organization (WHO), 60% of the global population and 80% of the population of developing countries prefer herbal drugs for their healthcare needs (Chikezie, 2015). The natural product or herbal medicine contains multiple chemical compounds with therapeutic and nutritional value, determining its multi-target nature (Zhong et al., 2015; Li Q. et al., 2021).

Various studies suggest that herbal medicines such as berberine, betulinic acid, celastrol, curcumin, salidroside, polydatin, and resveratrol could improve renal function and slow kidney disease progression (Zhong et al., 2015). Mitochondria are one of the primary organelles responsible for energy production. They are essential for the kidney to eliminate waste in the blood and control the signaling transduction process, cell proliferation, cell cycle, cell growth, cell death, water, and electrolyte balance (Sun et al., 2014; Sun et al., 2019; Li Q. et al., 2021; Tang et al., 2021). Mitochondrial dysfunction is associated with AKI and CKD pathogenesis (Li Q. et al., 2021; Tang et al., 2021). It results in the breakdown of adenosine triphosphate (ATP), excessive production of reactive oxygen species (ROS), and release of proapoptotic proteins such as cytochrome c, inducing cell injury through apoptosis, inflammation, fibrosis, and oxidative damage to DNA and proteins (Sun et al., 2014).

There is increasing evidence that natural products can protect the kidneys against various toxic stimuli by maintaining mitochondrial fitness (Martínez-Klimova et al., 2020; Li Q. et al., 2021). Consequently, phytoconstituents such as flavonoids, terpenoids, steroids, and fatty acids have been the subject of significant research to determine their possible nephroprotective effects (Basist et al., 2022). Because of their ability to manipulate oxidative, inflammatory, and apoptotic variables, they are almost universally recognized as potentially useful for treating kidney-related conditions. This review discusses the molecular mechanisms of action of natural products that improve mitochondrial fitness and the pathological changes induced by their treatment.

## Methods

A literature search was performed to collect original research articles published in English on therapeutic compounds for kidney disease and their mechanism of action in the Google Scholar, Web of Science, Scopus, and PubMed databases. Various search terms were used, including kidney disease, autophagy, apoptosis, natural compounds, kidney cancer, phytochemical, drug delivery system, targeted signaling pathway, and prospective role of kidney treatment. All figures were created using the Adobe Illustrator software (San Jose, CA, United States).

## Mitochondrial dysfunction mechanisms in kidney diseases

**FIGURE 1:**
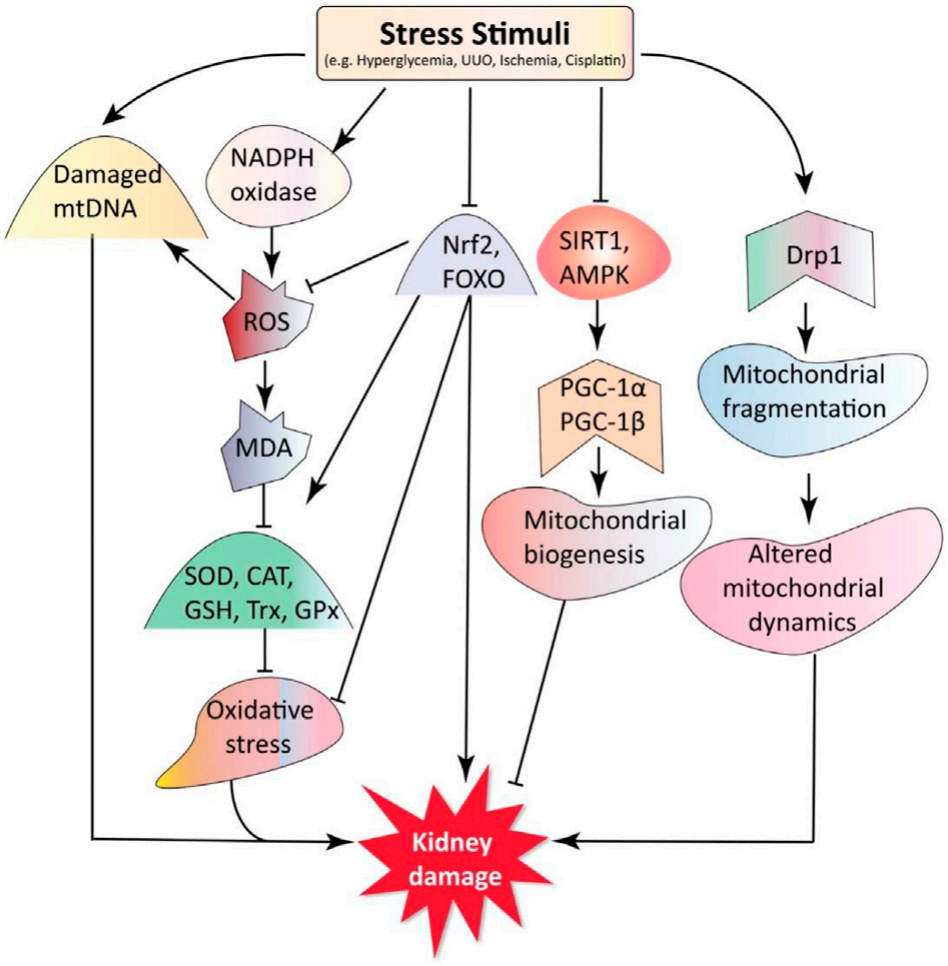
Mitochondria dysfunction in kidney diseases. Stress stimuli, such as hyperglycemia (HG), unilateral ureteric obstruction (UUO), cisplatin, and ischemia cause an imbalance between oxidative stress and antioxidants. Under stress stimuli, the expression of transcription factors nuclear factor erythroid 2-related factor 2 (*Nrf-2*) and forkhead box O (FOXO) decreases, activating nicotinamide adenine dinucleotide phosphate (NADPH) oxidase leading to excessive ROS and malondialdehyde (MDA) production. This oxidative stress also reduces the transcription of antioxidants such as superoxide dismutase (*SOD*), catalase (*CAT*), glutathione synthetase (*GSS*), glutathione peroxidase (*GPX*), and thioredoxin (*Trx*). Sirtuin 1 (SIRT1) and AMP-activated protein kinase (AMPK) can activate peroxisome proliferator-activated receptor γ coactivator 1α (PGC-1α), a master regulator of mitochondrial biogenesis. The activated dynamin-related protein 1 (Drp1) disturbs mitochondrial dynamics and causes mitochondria fragmentation. Moreover, various stress stimuli can induce mtDNA damage. Altogether, these cause changes in mitochondria structure and function, exacerbating kidney disease progression. Key: AMPK, AMP-activated protein kinase; CAT, catalase; DN, diabetic nephropathy; Drp1, dynamin-related protein 1; FOXO, forkhead-box class O; GSH, glutathione; GPx, glutathione peroxidase; GM, gentamycin; HN, hyperuricemic nephropathy; MDA, malondialdehyde; mtDNA, mitochondrial DNA; PGC-1α, peroxisome proliferator-activated receptor-γ coactivator 1α; ROS, reactive oxygen species; SIRT1, silent mating type information regulation 2 homolog 1; SOD, superoxide dismutase; Trx, thioredoxin; UUO, unilateral ureteral obstruction.

The kidney has the highest proportion of mitochondria of all tissues, providing the energy needed to maintain kidney functions, such as nutrient reabsorption, regulation of electrolytes and fluid balance, and acid-base homeostasis (Bhargava and Schnellmann, 2017). Mitochondrial DNA (mtDNA) health and the presence of mitochondrial biogenesis, mitochondrial dynamics, and oxidative stress is often required for mitochondria to perform their functions (Pedraza-Chaverri et al., 2016; Aparicio-Trejo et al., 2018). The mechanisms involved in mitochondrial dysfunction contributing to the pathogenesis of kidney diseases are discussed in the following sections (Figure 1).

Nitric oxide (NO) produced by endothelial nitric oxide synthase (eNOS) is believed to be responsible for protective effects such as the suppression of leukocyte and platelet activation and adhesion. Evidence supporting eNOS’s positive role includes lower *eNOS* expression in experimental models of glomerulonephritis and human biopsy specimens. This reduction in *eNOS* expression most likely reflects the necrosis of endothelial cells. The interaction of NO with superoxide anions or the myeloperoxidase (MPO) and hypochlorous acid (HOCL) system are two potential mechanisms that may lead to reduce NO production in acute glomerulonephritis (Heeringa et al., 2002).

Endothelial NO is an important vasodilator involved in various cardiovascular diseases (CVDs) and CKDs (Kumar et al., 2021). Renovascular hypertension is a leading cause of secondary hypertension, induced by atherosclerotic renovascular stenosis or fibromuscular dysplasia. Angiotensin II production, oxidative stress, and peroxynitrite generation reduce NO availability, causing hypertension, renal and endothelial dysfunction, and cardiac and vascular remodeling (Hiyoshi et al., 2005). eNOS uncoupling reduces NO availability in renovascular hypertension. NO donors and NO-derived metabolites reduce blood pressure and organ damage in experimental renovascular hypertension (Segawa et al., 2020). Therefore, understanding the function of reduced NO in renovascular hypertension stimulates the investigation of NO donors and molecules that can be converted into NO, which are potentially important to the future treatment of CVD and CKD (Pereira et al., 2022).

### Mitochondrial oxidative stress

Under normal physiological conditions, mitochondria serve as the primary intracellular location for the production of ROS and oxidants (Satoh et al., 2011; Xu et al., 2012). Through a process called the respiratory chain, mitochondria can produce ATP in Complex V, where a low concentration of superoxide anions is produced in Complexes I and III. While low ROS levels are important for cell function, high levels are toxic to mitochondria and cells. Mitochondrial damage can increase the release of cytochrome C into the cytosol, activating excessive ROS production and leading to oxidative stress and activation of the inflammatory response, resulting in CKD progression. In addition, reduced expression of antioxidant genes induces mitochondrial oxidative stress (Satoh et al., 2011; Xu et al., 2012; Hui et al., 2017). Excessive ROS can cause mtDNA damage, resulting in mutations in the next generation of mitochondria, decreasing the efficiency of the respiratory chain, reducing ATP production, and damaging proteins and lipids. In addition, ROS can induce cell apoptosis by causing cytochrome C release, leading to mitochondrial dysfunction (Ruiz et al., 2013).

Mitochondrial dysfunction is marked by an increase in mitochondrial ROS (mtROS), a decrease in matrix metalloproteinases (MMPs), and increases in calcium influx, damage, and release of cytochrome C (Zhou et al., 2021). Electrons that leak out of the electron transport chain (ETC) combine with oxygen to make O^2−^, which superoxide dismutases (SODs) catalyze into hydrogen peroxide (H_2_O_2_). Reduced MMP levels and mitochondrial calcium overload are important signs of mitochondrial dysfunction, which has a significant effect on oxidative stress, inflammation, neurodegeneration, and apoptosis (Rahman et al., 2020). Mutations and releases of mtDNA break down the mitochondrial respiratory chain, increase the mtROS levels, and speed up mitochondrial dysfunction (Tang et al., 2021). Therefore, mitochondrial dysfunction plays an important role in the development of AKI, aberrant kidney repair, and CKD.

Mitochondrial oxidative stress stimulates the glomerulosclerosis and pathogenesis of diabetic kidney disease (DKD) (Zhang et al., 2019; Ogura et al., 2020). Additionally, renal ischemia-reperfusion, hyperglycemia (HG), cis-diamminedichloroplatinum (cisplatin), cadmium (Cd), and gentamicin (GM) induce oxidative stress and contribute to kidney injuries through the production of excess ROS (Visnagri et al., 2015; Adil et al., 2016; Ortega-Domínguez et al., 2017; ALTamimi et al., 2021). Oxidative stress stimulates NO production by NOS. NO reacts with superoxide radicals and produces cytotoxic ROS such as peroxynitrite (ONOO^−^), which causes nitrosative stress and oxidizes lipids, DNA, and proteins, resulting in changes to cell signaling and oxidative damage leading to cell necrosis and apoptosis (Kim et al., 2018).

Additionally, NO is crucial in the control of kidney, cardiovascular, and metabolic systems, both in normal and diseased states, creating interest in the development of therapeutic technologies that can alter NO bioactivity (Bulboacă et al., 2021). Innovative pharmacological and nutritional approaches that increase NO bioactivity and reduce oxidative stress may represent viable therapeutics for preventing and treating kidney disease (Carlström, 2021).

Changes in mitochondrial structure and function lead to decreased ATP production capacity in aging cells due to mitochondrial dysfunction. However, while mitochondrial oxidative stress decreases with age (Satoh et al., 2011; Kim et al., 2018), inflammatory signaling molecules can stimulate the production of free radicals to enhance oxidative stress (Zhang et al., 2019; Huang et al., 2020). p66^Shc^ is a prooxidant that accumulates in the kidneys of diabetic patients that induces ROS production by increasing the expression of nicotinamide adenine dinucleotide phosphate (NADPH) oxidases (*NOXs*) and inhibiting the expression of the endogenous antioxidant enzymes, including manganese superoxide dismutase (*MnSOD*) and glutathione synthetase (GSS) (ALTamimi et al., 2021). NOXs are enzymes that primarily serve the purpose of catalyzing ROS production. Using NADPH as an electron donor, NOXs can catalyze the transfer of electrons from NADPH to oxygen, resulting in the production of either superoxide or H_2_O_2_ (Lee et al., 2020). Under normal conditions, the expression level of NADPH oxidase 4 (*NOX4*) is highest in kidney tubular cells but also present at lower levels in endothelial cells, cardiomyocytes, and other cell types (Lee et al., 2020).

Enzymes with antioxidant activity such as GSS, SODs, and catalase (CAT) function as free radical scavengers to protect cells against the harmful effect of ROS. ROS can also activate nuclear respiratory factor-like 2 (Nrf-2), which translocates to the nucleus and binds to antioxidant-responsive elements (AREs) to activate the transcription of genes encoding antioxidant enzymes, such as NADPH quinone oxidoreductase (*NQO-1*), heme oxygenase-1 (*HO-1*), *SOD1*, and *SOD2* (Kim et al., 2018). The forkhead-box class O (FOXO) family includes FOXO1 and FOXO3 and function as transcription factors that inhibit ROS production and lipid peroxidation by enhancing the expression of ROS scavenging and antioxidant enzymes such as thioredoxin (*Trx*), thioredoxin reductases (*TXNRDs*), *GSS*, *SODs*, *CAT*, glutathione peroxidases 1/2 (*GPX1*/*2*) (ALTamimi et al., 2021).

Endothelial dysfunction, inflammation, and oxidative stress are associated with CKD and CVD (Jin et al., 2021), where they have similar roles (Kumar et al., 2022). Both CVD and CKD are associated with endothelial dysfunction, inflammation, and oxidative stress (Streeter et al., 2013). As kidney disease progresses, as measured by the glomerular filtration rate (GFR) and the amount of protein in the urine, the risk of CVD and chronic renal failure increases (Stenvinkel et al., 2008), leading to ESRD, where therapies are required to replace the kidneys or even a kidney transplant (Ravarotto et al., 2018). Traditional and non-traditional risk factors have a significant effect on the life expectancy of CKD patients. The interconnectivity of molecular mechanisms in oxidative stress, inflammation, and endothelial dysfunction, is the primary shared factor in determining CKD, high blood pressure, CVD, and cardiovascular-renal remodeling (Wang and Gao, 2022). Therefore, summarizing our existing understanding and identifying prospective future research pathways and therapeutic options for intervention and our current state of knowledge would help treat oxidative stress-related CKD.

### Mitochondrial dysfunction

Mitochondrial homeostasis is tightly regulated by balancing mitochondrial biogenesis, fission, fusion, and mitophagy. These processes are required to maintain mitochondria structure and function, particularly mitochondrial energetics (Yuan et al., 2012; Zhang et al., 2019). Mitochondrial biogenesis is regulated by various transcriptional coactivators and corepressors. The peroxisome proliferator-activated receptor-γ coactivator (PGC) family includes 1α (PGC-1α) and 1β (PGC-1β) and PGC-1-related coactivator (PRC), which act as key upstream transcriptional regulators of mitochondrial biogenesis (Martínez-Klimova et al., 2020; Qin et al., 2020). PGC-1α is known to be a prominent regulator of oxidative phosphorylation, the tricarboxylic acid (TCA) cycle, and fatty acid metabolism in the kidneys (Svensson et al., 2016). Decreased mitochondrial biogenesis exacerbates oxidative stress in kidneys with unilateral ureteral obstruction (UUO) (Qin et al., 2019; Martínez-Klimova et al., 2020). Pan-peroxisome proliferator-activated receptors (PPARs) agonist bezafibrate was found to increase *PGC-1α* expression, leading to stimulation of mitochondrial biogenesis (Martínez-Klimova et al., 2020). Since PGC-1α is activated by signals with high-energy demands, it is mostly expressed in the kidney tissues as renal cells require much energy to perform normal functions (Qin et al., 2019; Martínez-Klimova et al., 2020). Other markers of mitochondrial biogenesis include transcription factor A of mitochondria (TFAM), nuclear factor erythroid 2-related factor 1 (Nrf-1), and Nrf-2 (Qin et al., 2019). Mitochondrial biogenesis is an extremely complex process requiring mtDNA replication and the synthesis, import, and incorporation of proteins and lipids into the existing mitochondrial reticulum. PGC-1α coactivates the transcriptional activity of Nrf-1, which binds to the TFAM promoter, a direct mediator of mtDNA replication (Yuan et al., 2012).

Histone deacetylase sirtuin 1 (SIRT1) has emerged as a critical regulator of mitochondrial function via energy-sensing pathways that stimulate mitochondrial biogenesis (Li Y. et al., 2021). Sirtuin 1 (SIRT1) and 3 (SIRT3) are protein deacetylases that play a role in regulating mitochondrial processes, including biogenesis (Ahn et al., 2008). Nicotinamide adenine dinucleotide (NAD^+^) has been found to activate SIRT1, which then activates its downstream targets, including PGC-1α (Whitaker et al., 2016). SIRT3 is specifically located in the mitochondria, and its activation also stimulates mitochondrial biogenesis (Kong et al., 2010). The suppression of PGC-1α by a mutant histone deacetylase 5 (HDAC5) causes morphological alterations to mitochondria and inhibits mitochondrial enzymes (Yuan et al., 2012). Reduced PGC-1α expression causes mitochondrial dysfunction, reduces cell viability, and induces cell apoptosis and dedifferentiation, resulting in kidney failure (Qin et al., 2020).

Recent studies have explored how sirtuins change and their roles in various kidney and heart diseases to identify sirtuin activators and inhibitors that may help prevent these diseases (Wang W. et al., 2021). Several compounds that target sirtuins are promising drug candidates for various kidney and heart diseases (Kumar et al., 2020). However, large, well-designed clinical trials are still required to determine their effectiveness and safety. Because SIRT1 deacetylates its targets with the assistance of coenzyme NAD^+^, it is connected to cellular energy metabolism and redox status through various signaling and survival pathways (Kitada et al., 2013). SIRT1 can control lipid metabolism, autophagy, blood pressure, and salt balance in the kidneys and decrease renal cell death, inflammation, and fibrosis (Chen et al., 2020). In addition, it can prevent renal cell apoptosis. Therefore, the activation of SIRT1 in the kidney may be a potential therapeutic target that can increase resistance to many of the causative factors contributing to renal disorder development, such as diabetic nephropathy (DN).

### Altered mitochondrial dynamics

Mitochondrial structure and morphology are crucial for maintaining optimal ATP production. Mitochondrial morphology is tightly regulated by a series of processes encompassing fission, fusion, and mitophagy (Qin et al., 2019; Zhang et al., 2020). Mitochondrial fission occurs at the specific sites where mitochondria interconnect with the endoplasmic reticulum (ER), and four adapter proteins, fission protein 1 (Fis1), mitochondrial fission factor (MFF), and the mitochondrial elongation factors 1 and 2 (Mief1 and Mief2), mediates the fission (Martínez-Klimova et al., 2020). Mitochondria undergo fission to alter their number and shape to adjust to fluctuations in metabolic demands. Mitochondrial fission is controlled by dynamin-related protein 1 (Drp1), which moves to the mitochondrial outer membrane (MOM) following phosphorylation at position S616. Drp1 binds to its receptors Fis1 and mitochondrial dynamics protein 49/51 (MiD49/51) and creating large oligomers, fragmenting mitochondrial tubules, and leading to mitochondrial fragmentation (Ni et al., 2017; Qin et al., 2019; Haschler et al., 2021).

In contrast, mitochondrial fusion preserves mitochondria from mitophagy, induces ATP production, regulates the appropriate mtDNA distribution, and increases mitochondria size and metabolic components (Molina-Jijón et al., 2016; Qin et al., 2019; Haschler et al., 2021). Fusion is controlled by anchor proteins mitofusin 1/2 (MFN1/2) and optic atrophy 1 (OPA1). MFN1/2 regulates mitochondrial outer membrane fusion, and OPA1 regulates mitochondrial inner membrane fusion (Molina-Jijón et al., 2016; Hui et al., 2017; Haschler et al., 2021). Various stressors, such as enhanced ROS production, minimized cellular ATP, and obstructions in cellular respiration caused by oxidative damage, can disrupt mitochondrial dynamics (Ni et al., 2017). During metabolic or environmental stresses such as ATP depletion (Molina-Jijón et al., 2016), mitochondrial dynamic balance switches to fission, characterized by a fragmented morphology, excess ROS production, and reduced energy metabolism and mitochondrial membrane potential (MMP) (Qin et al., 2019). Excessive mitochondrial fission and mitochondrial dysfunction in podocytes under metabolic or environmental stresses lead to DKD progression (Ni et al., 2017; Qin et al., 2019). Under hyperglycemic conditions, Drp1 interacts with Bcl-2-associated X-protein (Bax), resulting in the release of mitochondrial apoptotic proteins such as cytochrome C into the cytoplasm. These proapoptotic proteins then activate the caspase signaling cascade, leading to cellular apoptosis (Ni et al., 2017; Qin et al., 2019).

### Mitochondrial DNA damage

Increased levels of mtDNA damage have been associated with mitochondrial dysfunction and alteration of mitochondrial morphology in the kidney (Satoh et al., 2011; Yu et al., 2018). Mitochondrial fission, chemotherapeutic agent cisplatin, and HG have been found to stimulate excess ROS production, causing mtDNA damage (Xu et al., 2012). mtDNA fragmentation due to cisplatin is regulated by DNAse I and endonuclease G. After being passively shifted to nuclei, DNAse I initiates the breakdown of single DNA strands (ssDNA) that are more sensitive to endonuclease G digestion (Miller et al., 2010). HG disrupts the mitochondrial respiratory chain, leading to MMP hyperpolarization, reduced mtDNA content, and decreased ATP production (Xu et al., 2012). Oxidative stress causes mtDNA damage (Qin et al., 2019), and the oxidative damage of mtDNA is associated with the aging phenotype in renal cells (Satoh et al., 2011). mtDNA mutation can also exacerbate ROS production due to impaired oxidative phosphorylation (OXPHOS), eventually leading to mitochondrial dysfunction (Wang D.-W. et al., 2021).

## Therapeutic effects of phytochemicals against mitochondrial dysfunction in kidney diseases

**FIGURE 2:**
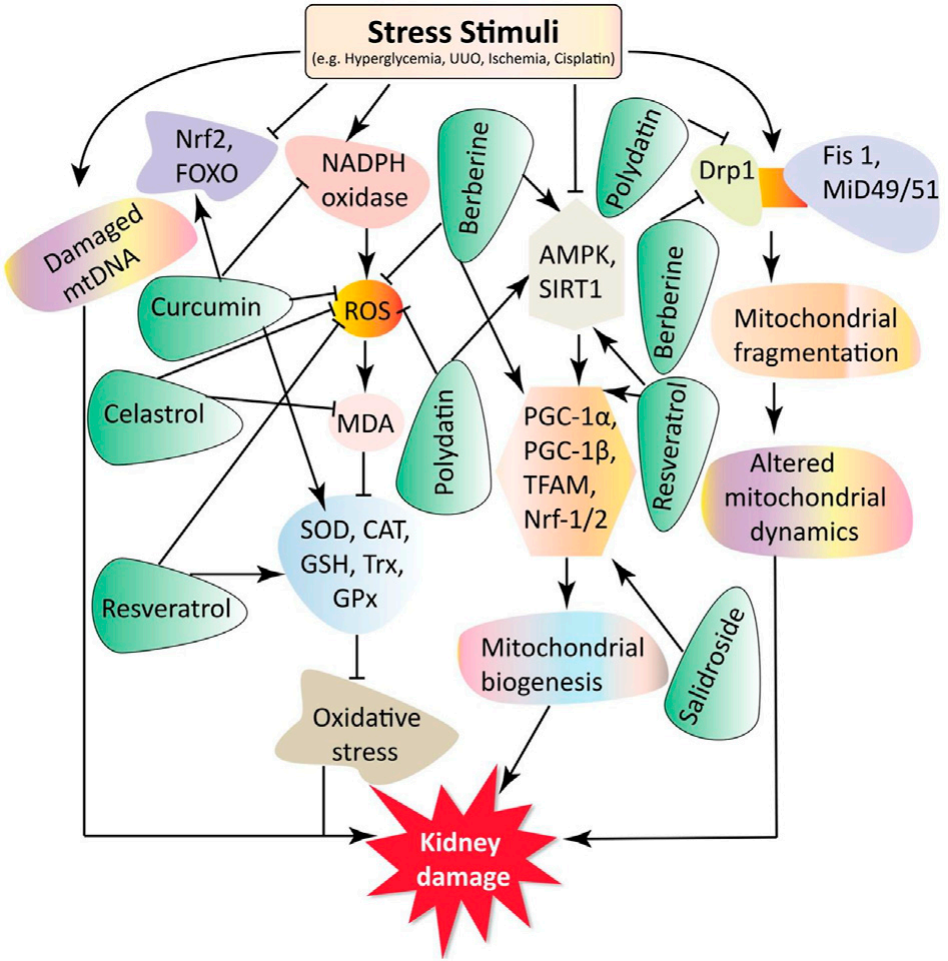
This schematic representation indicates that stress stimuli like HG, UUO, cisplatin, and ischemia regulate various pathological conditions involving oxidative stress, mitochondrial biogenesis, mtDNA damage, and altered mitochondrial dynamics. These ultimately lead to renal damage. The small molecule rotenone reduces mtDNA damage and ROS production. In addition, celastrol reduces the ROS and MDA oxidative stress markers. Polydatin, berberine, and resveratrol decrease ROS production. Salidroside increases *SIRT1* and *PGC-1α* expression, resulting in mitochondrial biogenesis, enhancing and protecting the kidneys. Curcumin and resveratrol increase the transcription of antioxidants markers *SOD*, *CAT*, *GSS*, *GPX*, and *Trx*. Moreover, berberine and resveratrol increase the expression of mitochondrial biogenesis markers *PGC-1α*, *TFAM*, *Nrf-1*, and *Nrf-2*. Furthermore, polydatin decreases the expression of mitochondrial fission protein *Drp1* and preserves kidney function. Key: DN, diabetic nephropathy; Drp1, dynamin-related protein 1; GSH, glutathione; GPx, glutathione peroxidase; GM, gentamycin; HN, hyperuricemic nephropathy; MDA, malondialdehyde; mtDNA, mitochondrial DNA; Nrf-1 and Nrf-2, nuclear respiratory factors 1 and 2; PGC-1α, peroxisome proliferator-activated receptor-γ coactivator 1α; ROS, reactive oxygen species; SIRT1, silent mating type information regulation 2 homologs 1; SOD, superoxide dismutase; Trx, thioredoxin; TFAM, transcription factor A of mitochondria.

Mitochondrial dysfunction plays a critical role in CKD progression, including DN, ON, kidney aging, drug-induced AKI, ischemia-reperfusion injury (IRI)-induced AKI, and chemical-induced AKI. Some small molecule natural products have been reported to mediate renoprotection and improve the various types of CKD. Here, we provide a comprehensive overview of the protective effects of small molecule natural products against mitochondrial dysfunction in the kidney. The experimental and disease models, pathobiology involved, protective effects, and molecular markers altered by these compounds are summarized in Tables 1, 2 and Figure 2.

**TABLE 1:** Kidney protective effects are provided by phytochemicals targeting mitochondrial fitness in animal models.

Disease model	Phytochemical	Dosage	Experimental model	Pathobiology	Alteration in molecular markers in response to treatment	References
DN	Berberine	300 mg/kg/d, 8 w	db/db diabetic mice	Mesangial matrix accumulation	↓TG, ↓ACR, ↓FBG, ↓FFA, ↓Drp1	Qin et al. (2019)
		200, 300 mg/kg/d, 8 w	db/db mice model	Lipid accumulation	↓FFA, ↓TG	Qin et al. (2020)
	Curcumin	100 mg/kg, 12 w	Streptozotocin (STZ)-injected rats	Fibrosis, inflammation, oxidative stress, mitochondrial dysfunction	↓Collagen I/III, ↓TGF-β1, ↓NF-κB, ↓ROS, ↓MDA ↑GSH↑, ↑MnSOD, ↓cytochrome-C, ↓caspase-3, ↑Bcl-2↑, ↑Nrf-2, ↓p^66^Shc	ALTamimi et al. (2021)
	Polydatin	100 mg/kg, 8 w	KKAy mice	Mitochondrial dysfunction	↓Drp1	Ni et al. (2017)
	Resveratrol	30 mg/kg/d, 12 w	STZ-injected CD-1 mouse	Oxidative stress, apoptosis, mitochondrial dysfunction	↑MnSOD, ↓MDA, ↓caspase 3 ↑SIRT1, ↑PGC-1α, ↑Nrf-1↑, ↑TFAM	Zhang et al. (2019)
	Salidroside	50, 100 mg/kg/d, 10 w	High-fat diet (HFD)/STZ-induced diabetic rats	Extracellular matrix (ECM) deposition, mitochondrial dysfunction	↓collagen I, ↓FN, ↓α-SMA ↑SIRT1, ↑PGC-1α	Xue et al. (2019)
Obstructed nephropathy	Resveratrol	12.5 or 25 mg/kg, 14 d	UUO-operated C57BL/6 mice	Fibrosis	↓Collagen, ↓TGF-β/Smad, ↓FN, ↓α-SMA, SIRT1↑	Liu et al. (2019)
Aged kidney	Resveratrol	40 mg/kg, 6 m	18-month-old C57BL/6 mice	ECM accumulation, Apoptosis, Oxidative stress, mitochondrial dysfunction	↓Col IV, ↓TGF-β1, ↑COX IV, ↑BCL-2, ↓Bax, ↑Nrf-2, ↑HO-1, ↑NQO-1, ↓ROS, ↑SOD, ↑SOD2, ↑SIRT1, ↑AMPK, ↑PGC-1α	Kim et al. (2018)
Sepsis-induced AKI	Curcumin	4 mg/kg, 1, 6, 12 and 24 h	Sepsis-induced AKI mice model	Inflammation, Oxidative stress	↓IL-6, ↓ TNF-*α* ↓ROS, ↓MDA, ↑GSH, ↑SOD	Wang et al. (2021a)
	Polydatin	30 mg/kg, 6, 12, 18 h	Rats with caecal ligation and puncture (CLP)-induced sepsis	Mitochondrial dysfunction, inflammation	↑Mt membrane potential, ↑Mt-ATP levels ↓IL-6, ↓LPO	Gao et al. (2015)
		30 mg/kg, 12 h	C57BL/6 mice with CLP-induced sepsis	Mitochondrial dysfunction, inflammation, apoptosis	↓KIM-1, ↓NLRP3, ↓IL-6, ↓TNF-*α*, ↓IL-1β, ↓caspase-1, ↓Bax, ↓caspase-3 ↑ SIRT1, ↑Bcl-1	Gao et al. (2020)
	Resveratrol	0.3 ml; 50 mg/kg, 30 min	Rats with CLP-induced sepsis	Mitochondrial dysfunction	↑SIRT1/3, ↑ATP, ↓cytochrome-C, ↑SOD2	Xu et al. (2016)
Ischemia-reperfusion-induced AKI	Berberine	20 and 40 mg/kg, 4 w	Renal ischemia/reperfusion (RIR)-induced Wistar rats	Oxidative stress	↑SOD, ↑GSH, ↓MDA	Kaur et al. (2016)
				Mitochondrial dysfunction apoptosis	↓KIM-1	
				Inflammation	↑Bcl-2, ↓caspase-3, ↓Bax	
					↓TNF-*α*	
Drug-induced AKI	Berberine	10, 20, and 40 mg/kg; p.o., 7 d	Gentamycin–induced nephrotoxicity Sprague-Dawley rats	Oxidative stress, inflammation, apoptosis, mitochondrial dysfunction	↓MDA, ↑SOD, ↑GSH	Adil et al. (2016)
					↓NF-κB	
					↑Bcl-2	
					↑mt complex (I – IV), ↓NO, ↓KIM-1	
	Celastrol	1, 2 mg/kg/d	Cisplatin-induced C57BL/6 mice	Oxidative stress, apoptosis, inflammation, mitochondrial dysfunction	↓MDA	Yu et al. (2018)
					↓Bax, ↑Bcl-2	
					↓IL-1β, ↓IL-6, ↓TNF-*α*, ↓ NF-κB p65	
					↓KIM -1, ↓NGAL	
	Curcumin	200 mg/kg/d, 3 d	Cisplatin-induced male Wister rats	mitochondrial dysfunction and dynamics	↑SIRT3, ↑mt complex I, ↑OXPHOS	Ortega-Domínguez et al. (2017)
					↓Fis1	
					↑OPA1, ↑MFN1	
Chemical-induced AKI	Curcumin	400 mg/kg, 5 d	Maleate-induced Wistar rats	Oxidative stress	↓MDA, ↓ROS, ↑GSSG, ↑GSH	Molina-Jijón et al. (2016)
				Mitochondrial fragmentation	↓Drp1, ↓Fis1	
		400 mg/kg, 10 d	Chromium-induced Wistar rats	Oxidative stress	↑CAT, ↑GR, ↑GPx, ↑SOD, ↑GSH, ↑GST	Molina-Jijón et al. (2011)
				Mitochondrial dysfunction	↑mt complex (I – IV)	
	Resveratrol	400 mg/kg, 90 d	Cadmium (Cd)-induced white chickens	Oxidative stress, mitochondrial dysfunction	↑T-SOD, ↑Cu-Zn SOD, ↑CAT, ↑GST, ↑GSH-Px, ↓MDA, ↑Nrf-2	Zhang et al. (2020)
					↑SIRT3, ↑SIRT1, ↑PGC-1α, ↑Nrf-1, ↑TFAM	
		400 mg/kg, 14 d	Aldosterone-induced C57BL/6J mice	Mitochondrial dysfunction	↑PGC-1α, ↑TFAM, ↑SIRT-3	Yuan et al. (2012)
Other AKI-related diseases	Curcumin	60 mg/kg/day, 7 d	Nephrectomy-induced Wistar rats	Oxidative stress, mitochondrial dysfunction	↑CAT, ↑SOD, ↑GR, ↑GPx, ↑GST, ↑Nrf-2	Ortega-Domínguez et al. (2017)
					↓Drp1, ↓Fis1, ↑mt complex I/V	
	Resveratrol	30 mg/kg, 1 h	Hemorrhagic shock-induced Evans rats	Oxidative stress, mitochondrial dysfunction	↑SOD2, ↑CAT, ↑Nrf-2, ↓ROS ↓NGAL, ↓LP, ↓NADH, ↑SIRT1, ↑PGC1-α	Wang et al. (2015)

ATP, adenosine triphosphate; AMPK, AMP-activated protein kinase; ACR, microalbumin-to-creatinine ratios; BCL-2, B-cell lymphoma 2; CAT, catalase; Col IV, Collagen IV; Cr, Chromium; Cd, cadmium; COX IV, Cytochrome Oxidase IV; Drp1, dynamin-related protein 1; ECM, Extracellular matrix; ERRAα, Estrogen-related receptor alpha; FBG, fasting blood glucose; FAO, fatty acid oxidation; FN, fibronectin; FFA, free fatty acids; Fis1, mitochondrial fission protein 1; GSSG, glutathione disulfide; GPx, glutathione peroxidase; GSH, glutathione; HO-1, Heme oxygenase-1; ICAM-1, intercellular adhesion molecule-1; IL-6, interleukin 6; IL-1β, interleukin 1 β; KIM-1, kidney injury molecule-1; Mid51 and Mid49, mitochondrial dynamics proteins of 51 and 49 kDa; MMP, mitochondrial membrane potential; mtDNA, mitochondrial DNA; MFF, mitochondrial fission protein; MnSOD, manganese superoxide dismutase; MMP-9, matrix metalloproteinase-9; MDA, malondialdehyde; NGAL, neutrophil gelatinase associated lipocalin; NQO-1, Quinone Oxidoreductase 1; NADH, nicotinamide adenine dinucleotide plus hydrogen; Nrf-1 and Nrf-2, nuclear respiratory factors 1 and 2; NF-κB, nuclear factor-kappa B; OXPHOS, oxidative phosphorylation; PPAR, peroxisome proliferator-activated receptor; PA, palmitic acid; PGC-1α, peroxisome proliferator-activated receptor-γ co-activator 1α; RIR, renal ischemia reperfusion; ROS, reactive oxygen species; STZ, streptozotocin; SIRT1, silent mating type information regulation 2 homolog 1; SOD1 and SOD2, superoxide dismutase 1 and 2; TBARS, thiobarbituric acid reactive substances; TFAM, transcription factor A; TG, triglyceride; TNF-*α*, tumor necrosis factor α; TGF-β1, transforming growth factor- β-1; UUO, unilateral ureteral obstruction; α-SMA, α-smooth muscle actin.

**TABLE 2:** Kidney protective effects are provided by phytochemicals targeting the mitochondrial fitness in cellular models.

Disease model	Phytochemical	Dosage	Experimental model	Pathobiology	Alteration in molecular markers in response to treatment	References
DN	Berberine	0.4 μM/L, 12 h	Podocytes cells treated with PA	Mitochondrial dysfunction, oxidative stress, apoptosis	↓MMP-9, ↓Drp1, ↓MFF, ↓Fis1, ↓Mid49, ↓Mid51	Qin et al. (2019)
					↓ROS, ↓MDA	
					↑PGC-1α, ↑TFAM↑, ↑Nrf-1, ↑Nrf-2	
					↓caspase-3, ↓Bax, ↓cytochrome C	
					↑Bcl-2	
		0.4 μM/L, 12 h	Podocytes cells treated with PA	Oxidative stress, mitochondrial dysfunction, lipid accumulation	↓ROS, ↓MDA, ↑SOD	Qin et al. (2020)
					↑AMPK, ↑PGC-1α, ↑OXPHOS, ↑FAO	
					↓FFA, ↓TG	
	Polydatin	25 mM, 24, 48, 72 h	HG-induced MPC5 cells	Apoptosis, mitochondrial fragmentation, dysfunction, oxidative stress	↓Caspase 3, 9, ↓cytochrome-C	Ni et al. (2017)
					↓Drp1	
					↓ROS	
	Resveratrol	10 μM, 6 h	Rat mesangial cells treated with high glucose	Oxidative stress, mitochondrial dysfunction	↑MnSOD, ↓ROS	Xu et al. (2012)
					↑mt complex III, ↑mtDNA	
					↑ATP	
		10 μM/L, 48 h	Podocytes exposed to high glucose	Oxidative stress, mitochondrial dysfunction	↓mtROS	Zhang et al. (2019)
					↑mt respiratory chain complex I/III	
Obstructed nephropathy	Resveratrol	5–20 μM, 72 h	HK-2 cells treated with TGF-β	Fibrosis	↓FN, ↓α-SMA, ↓TGF-β1	Liu et al. (2019)
					↓p-Smad3	
Aged kidney	Resveratrol	50µM, 24 h	H_2_O_2_-induced HK2 cells	Oxidative Stress	↑HO-1, ↑NQO-1, ↑SOD1, ↑SOD2	Kim et al. (2018)
Sepsis-induced AKI	Curcumin	0.3684 g, 12 d	LPS-induced HK-2 cells	Oxidative stress	↓ROS	Wang et al. (2021a)
Drug-induced AKI	Celastrol	10–100 μM, 24 h	Cisplatin-induced HK-2 and RTECs	Mitochondrial dysfunction, inflammation, apoptosis	↓KIM-1, ↑mtDNA, ↑MMP	Yu et al. (2018)
					↓IL-1β, ↓IL-6, ↓COX-2, ↓NF-κB, ↓ROS	
					↑Bcl -2, ↓Bax, ↓Caspase-3	
Chemical-induced AKI	Resveratrol	50 μM/L, 30 min	Aldosterone induced podocytes	Mitochondrial dysfunction	↑mtDNA, ↑PGC-1α, ↑TFAM, ↑SIRT3	Yuan et al. (2012)
				Apoptosis	↓caspase-9, ↓caspase-3	

AMPK, AMP-activated protein kinase; BCL-2, B-cell lymphoma 2; Drp1, dynamin-related protein 1; FAO, fatty acid oxidation; FN, fibronectin; FFA, free fatty acids; Fis1, mitochondrial fission protein 1; HO-1, Heme oxygenase-1; HK-2, human renal proximal tubule epithelial cell line; HG, hyperglycemia; Mid51 and Mid49, mitochondrial dynamics proteins of 51 and 49 kDa; MMP, mitochondrial membrane potential; mtDNA, mitochondrial DNA; MFF, mitochondrial fission protein; MnSOD, manganese superoxide dismutase; MMP-9, matrix metalloproteinase-9; MDA, malondialdehyde; NQO-1, Quinone Oxidoreductase 1; Nrf-1 and Nrf-2, nuclear respiratory factors 1 and 2; OXPHOS, oxidative phosphorylation; PA, palmitic acid; PGC-1α, peroxisome proliferator-activated receptor-γ co-activator 1α; ROS, reactive oxygen species; SIRT3, silent mating type information regulation 2 homolog 3; SOD1 and SOD2, superoxide dismutase 1 and 2; TFAM, transcription factor A; TG, triglyceride.

### Diabetic nephropathy

DN is a microvascular complication of diabetes that occurs in patients with type 1 and type 2 diabetes (Kwon et al., 2017; Ni et al., 2017; Sohn et al., 2017; Huang et al., 2020) that leads to mitochondrial dysfunction in podocytes (Qin et al., 2019; Qin et al., 2020; ALTamimi et al., 2021). Podocytes are terminally differentiated and highly specialized glomerular epithelial cells that contain high numbers of mitochondria (Ni et al., 2017; Qin et al., 2019). Damaged podocytes act as a marker of DKD progression (Ni et al., 2017; ALTamimi et al., 2021). HG and lipotoxicity during diabetes are known to contribute to mitochondrial dysfunction (Zhang et al., 2019). ROS production, mostly of mitochondrial origin, induces DN initiation and development in patients. NOX and uncoupling of eNOS are the other sources of ROS in diabetic patients (Zhang et al., 2019; ALTamimi et al., 2021). Administration of berberine to *db/db* mice decreased free fatty acid (FFA) and triglyceride (TG) levels and attenuated pathohistological changes, such as basement membrane thickening, mesangial expansion, and glomerulosclerosis, resulting in protection against podocyte apoptosis (Qin et al., 2019). In cultured mouse podocytes exposed to palmitic acid (PA), berberine treatment was found to suppress the activation of Drp1-mediated mitochondria fission. Therefore, mitochondrial ROS production, dysfunction, and fragmentation were attenuated with berberine treatment (Qin et al., 2019). Furthermore, berberine was also shown to protect glomerular podocytes against PA-induced oxidative damage by increasing SOD activity and reducing excessive mtROS and MDA. It also preserves mitochondrial function by activating AMP-activated protein kinase (AMPK), enhancing *PGC-1α* expression. Therefore, berberine treatment was shown to induce mitochondrial biogenesis and restore oxidative phosphorylation and fatty acid oxidation (FAO) (Qin et al., 2020).

Curcumin treatment was also found to protect the kidneys against streptozotocin (STZ)-induced diabetic injury. Curcumin also restores the ROS-antioxidant balance under diabetic stress conditions. Furthermore, it decreased ROS levels, reduced the nuclear activity of the nuclear factor-kappa B (NF-κB), downregulated protein kinase CβII (PKCβII), NOX, and p66^Shc^, and decreased the activation of p66^Shc^. Moreover, it increased the transcript level of *MnSOD* and *GSS*, the protein levels of B-cell lymphoma-2 (Bcl-2) and MnSOD, and the nuclear levels of Nrf-2 and FOXO3A (ALTamimi et al., 2021). Curcumin also lowered adenine-induced high blood pressure, urinary albumin, the inflammatory cytokines interleukins 1 (IL-1) and 6 (IL-6), tumor necrosis factor α (TNF-α), cystatin C (CST3), and adiponectin (ADIPOQ) (Kumar et al., 2021). In addition, it increased sclerostin levels in the blood and reduced oxidative stress in kidney homogenates (Ali et al., 2018). This study also showed that curcumin had anti-hyperuricemic and anti-inflammatory effects by stopping the activation of the NLR family pyrin domain containing 3 (NLRP3) inflammasome in the kidney (Chen et al., 2019). Curcumin also makes the intestinal alkaline phosphatase and tight junction proteins more active and fixes the leakiness of the gut. This action lowers the number of inflammatory biomolecules in the blood, and curcumin in the intestine may explain its low bioavailability, which may have anti-inflammatory effects *in vivo* and benefit CKD (Ghosh et al., 2014). Therefore, curcumin could potentially be used to treat hyperuricemia and the inflammation associated with renal treatment.

Polydatin was also found to attenuate mitochondrial fragmentation by suppressing Drp1 activity in KKAy mice. Polydatin also inhibited podocyte apoptosis by suppressing Drp1 and attenuating mitochondrial dysfunction and ROS production in HG-induced MPC5 cells (Ni et al., 2017). Resveratrol treatment in podocytes exposed to high glucose resulted in conserved mitochondrial function, reduced excessive mitochondrial ROS production, improved respiratory chain complex I and III activity, and inhibited the release of cytochrome C (Zhang et al., 2019). In diabetic mice, resveratrol treatment decreased MDA levels and increased MnSOD activity in the renal cortex. In addition, resveratrol mechanistically protected glomerular podocytes by enhancing SIRT1/PGC-1α signaling, suppressing mitochondrial oxidative stress in the diabetic milieu (Zhang et al., 2019). In high glucose-treated rat mesangial cells, resveratrol treatment was also shown to increase MnSOD activity and decrease mitochondrial complex III activity. Consequently, mitochondrial function was improved, indicated by restored MMP, ATP production, and mtDNA content (Xu et al., 2012). Finally, salidroside treatment in STZ-induced mice maintained mitochondrial function by increasing mtDNA copy number and SIRT1 and PGC-1α expression, inhibiting kidney fibrosis (Xue et al., 2019).

### Obstructed nephropathy

The common pathogenesis of obstructed nephropathy involves fibrosis, inflammation, and apoptosis (Manucha and Valles, 2012; You et al., 2019). Fibrosis refers to extracellular matrix (ECM) accumulation in glomerular and tubulointerstitial tissue, inducing renal dysfunction. It involves multiple signaling pathways such as transforming growth factor β (TGF-β) and suppressor of mothers against decapentaplegic (Smad) and the assembly of fibrotic markers such as collagen I, fibronectin (FN), and α-smooth muscle actin (α-SMA) (Liu et al., 2019; You et al., 2019). Mice with UUO are a common model of progressive CKD, characterized by the development of tubulointerstitial fibrosis (Manucha and Valles, 2012; You et al., 2019; Martínez-Klimova et al., 2020). During renal injury, the injured tubular cells are associated with interstitial macrophages and myofibroblasts that produce cytokines such as TNF-α, interleukin 1β (IL-1β), and intercellular adhesion molecule-1 (ICAM-1) that induce an inflammatory response in the kidney (Manucha and Valles, 2012). Mitochondria-derived oxidative stress also induces an inflammatory response in obstructive kidney disease (Sun et al., 2014). Mitochondrial dysfunctions or abnormalities are observed in obstructed kidneys, resulting in inflammation exacerbation, oxidative stress, and eventually kidney fibrosis development (Sun et al., 2014; Martínez-Klimova et al., 2020).

Interestingly, a study showed that the renoprotection effect of resveratrol occurred at specific concentrations. Low concentrations of resveratrol (5–20 μM) inhibited pro-fibrotic signaling and improved renal function by increasing *SIRT1* expression in the TGF-β-induced human tubular epithelial cell (TEC) cell line HK-2. However, these protective effects were not observed with higher concentrations of resveratrol (≥40 μM), which induced mitochondrial dysfunction by increasing ROS and mtROS levels and decreasing mitochondrial length and density, ATP production, and *PGC-1α* expression (Liu et al., 2019). Consistent with the *in vitro* results, the renoprotective effects of resveratrol treatment in UUO-operated C57BL/6 mice were observed at lower doses (≤25 mg/kg). However, higher concentrations of resveratrol enhanced pro-fibrotic factors and reduced *TFAM* expression (Liu et al., 2019).

### Kidney aging

Age-related renal changes represent glomerulosclerosis, interstitial fibrosis, arteriosclerosis, and tubular atrophy (Lee et al., 2019), resulting from various potential biological aging processes, including the expression of senescence genes, hormonal changes, increased oxidative stress, and mitochondrial damage (Kim et al., 2018). Normal kidney aging is associated with the slow development of functional and structural changes (Satoh et al., 2011). The inactivation of SIRT1 and the activation of the renin-angiotensin system, oxidative stress, and mitochondrial dysfunction are associated with aging (Kim et al., 2018). A recent study has assessed the potential use of resveratrol in delaying kidney aging (Uddin et al., 2021). Administration of resveratrol at 40 mg/kg for 6 months in 24-month-old C57BL/mice prevented aging-related kidney damage by activating Nrf-2 and SIRT1 signaling and ROS suppression. Resveratrol treatment also prevented oxidative stress by increasing SOD levels in H_2_O_2_-induced HK2 cells (Kim et al., 2018).

### Drug-induced acute kidney injury

Certain drugs can alter intraglomerular hemodynamics and activate inflammation in renal tubular cells, resulting in AKI and tubulointerstitial disease (Shahrbaf and Assadi, 2015). Gentamycin (GM) is widely recognized as an aminoglycoside antibiotic that can induce nephrotoxicity (Cui et al., 2019). GM activates the intrinsic apoptosis pathway by stimulating the release of cytochrome C. GM also enhances oxidative stress by increasing the production of superoxide anion and hydroxyl radicals and lowering the expression of antioxidant genes such as *SOD*, *GSS*, and mitochondrial enzymes NADH dehydrogenase and cytochrome C oxidase. Moreover, GM inhibits respiratory complex I and IV and ATP synthesis, inducing mitochondrial dysfunction (Adil et al., 2016; Cui et al., 2019; Khaksari et al., 2021). Berberine treatment in GM-induced nephrotoxicity in rats significantly increased *SOD*, *GSS*, and *Bcl-2* expression and mitochondrial enzyme activity. Berberine also reduced ROS and MDA levels and inflammatory and tubular injury markers such as kidney injury molecule-1 (KIM-1), neutrophil gelatinase-associated lipocalin (NGAL), and NF-κB in GM-induced mice (Adil et al., 2016).

Cisplatin nephrotoxicity is commonly characterized by oxidative stress, inflammation, and apoptotic cell death in tubules (Miller et al., 2010). Cisplatin alters mitochondrial bioenergetics by lowering mitochondrial oxygen consumption and ATP levels, which are associated with oxidative stress-induced mtDNA damage. Moreover, cisplatin disrupted mitochondria dynamics, indicated by induced *Fis1* and decreased *SIRT3* expression (Ortega-Domínguez et al., 2017). GM-induced oxidant-antioxidant imbalance also altered membrane lipid composition *via* lipid peroxidation, indicated by increased MDA levels (Randjelovic et al., 2017). Celastrol treatment in GM-induced nephrotoxicity improved mitochondrial function, reduced oxidative stress, and attenuated tubular injury, apoptosis, and inflammation (Yu et al., 2018). Furthermore, celastrol was found to improve mitochondrial function by maintaining MMP and OXPHOS activities in cisplatin-treated renal tubular epithelial RTC cells (Yu et al., 2018). Another phytochemical, curcumin, may also prevent nephrotoxicity by upregulating *Fis1* and *SIRT3* in cisplatin-induced Wister rats (Ortega-Domínguez et al., 2017).

### Sepsis-induced acute kidney injury

Sepsis refers to uncontrolled and adverse host reactions to microbial infection and is a leading cause of mortality and complex illness worldwide (Xu et al., 2016). Sepsis progression is accompanied by multiple organ dysfunction (Gao et al., 2015; Xu et al., 2016). During sepsis-induced AKI (SI-AKI), cytokine release, oxidative stress, and apoptosis are major pathological features in the development of organ dysfunction (Manucha and Valles, 2012; Gao et al., 2015, 2020; Uddin et al., 2018). The kidney is affected during sepsis, and AKI is a general feature during sepsis pathogenesis which is associated with high mortality rates (Xu et al., 2016; Gao et al., 2020; Wang D.-W. et al., 2021). In response to sepsis, renal tubular cells have decreased oxygen consumption, indicating extreme mitochondrial dysfunction (Xu et al., 2016). Mitochondrial dysfunction increases ROS production in renal tubular epithelial cells during AKI progression. Mitochondrial outer membranes deteriorate, and mitochondrial edema occurs, resulting in proapoptotic cytochrome C release and eventually cellular apoptosis activation (Manucha and Valles, 2012; Wang D.-W. et al., 2021).

In lipopolysaccharides (LPS)-induced HK-2 cells, curcumin treatment restored mitochondrial function and decreased ROS levels (Wang D.-W. et al., 2021). In addition, curcumin treatment in SI-AKI mice suppressed the activation of inflammatory cytokines and enhanced mitochondrial protection by attenuating ROS and MDA levels and increasing GSS and SOD activity (Wang D.-W. et al., 2021), while resveratrol treatment improved mitochondrial function by increasing *SIRT1* and *SIRT3* expression and ATP production. Moreover, resveratrol treatment suppressed mitochondrial cytochrome c and upregulation of SOD2, contributing to the inhibition of tubular apoptosis (Xu et al., 2016).

Polydatin treatment also decreased mitochondrial dysfunction in the sepsis-induced rat model (Gao et al., 2015). Another mechanistic study in sepsis-induced C57BL/6 mice showed that polydatin-induced Parkin translocation and mitophagy were mediated by SIRT1. Moreover, mitochondrial dysfunction, mitochondrial-dependent apoptosis, and NLRP3 inflammasome activation were attenuated in polydatin-treated mice (Gao et al., 2020).

### Ischemia-reperfusion-induced acute kidney injury

Renal ischemia-reperfusion is the most common cause of AKI (Haschler et al., 2021), and its pathophysiology includes renal vasoconstriction, inflammation, apoptosis, tubular and glomerular damage (Visnagri et al., 2015), and mitochondrial dysfunction (Haschler et al., 2021). The deteriorating effects of ischemia-reperfusion on mitochondria include increased ROS production, decreased antioxidants, modification of pyridine nucleotide ratios, fluctuating Ca^2+^ concentration, and enhanced inorganic phosphate in the matrix (Visnagri et al., 2015). MMP and the ATP pool are the major determinants of mitochondrial architecture, which are decreased during ischemia, inducing mitochondrial fragmentation (Haschler et al., 2021). In renal ischemia-reperfusion (RIR)-induced Wistar rats, berberine treatment protected kidneys against oxidative stress and mitochondrial dysfunction. Moreover, upregulated anti-apoptotic protein Bcl-2 and downregulated apoptotic proteins caspase-3 (Casp3) and Bax were found in berberine-treated mice (Visnagri et al., 2015).

### Chemical-induced acute kidney injury

Chemical-induced AKI has emerged as a significant concern (Li et al., 2019) Maleate-induced nephrotoxicity is associated with ATP breakdown, enhanced ROS production, and GSH depletion. Curcumin treatment in maleate-induced Wistar rats suppressed oxidative stress and attenuated mitochondrial fragmentation by downregulating Drp1 and Fis1 (Molina-Jijón et al., 2016). Furthermore, curcumin treatment prevented potassium dichromate (K_2_Cr_2_O_7_)-induced mitochondrial dysfunction by increasing MMP, complexes I-III and V, and CAT, glucocorticoid receptor (GR), GPX, SOD, GSS, and glutathione S-transferase (GST), reducing oxidative stress (Molina-Jijón et al., 2011).

Cadmium (Cd) is a toxic metallic compound that can accumulate in the kidney. Cd accumulates across the renal cortex and medulla, inducing kidney injury. Oxidative stress occurs due to acute or subchronic Cd action, impairing the antioxidant defense system and causing nephrotoxicity. Cd exposure also induces mitochondrial structural damage and MMP loss. Resveratrol treatment suppressed Cd-induced oxidative stress and upregulated mitochondrial function-related factors SIRT3, SIRT1, PGC-1α, Nrf-1, and TFAM. Furthermore, resveratrol ameliorated excessive mitochondria fission and enhanced mitochondria fusion, suppressing PTEN-induced kinase (PINK1)/Parkin-mediated mitophagy (Zhang et al., 2020).

The mineralocorticoid hormone aldosterone mediated nephrotoxicity through upregulation of mitochondria ROS production and reductions in MMP, ATP production, and mtDNA copy number. Aldosterone also induces CASP3 and caspase 9 (CASP9) mediated apoptosis in aldosterone-instigated podocytes (Yuan et al., 2012). Administration of 400 mg/kg resveratrol for 14 days in aldosterone-induced C57BL/6J mice showed improvement in mitochondria function. Treatment with 50 μM/L resveratrol for 30 min in aldosterone-induced podocytes increased mtDNA copy number and *PGC-1α*, *TFAM*, and *SIRT3* expression. Apoptotic proteins CASP3 and CASP9 were also downregulated in response to the treatment (Yuan et al., 2012).

### Therapeutic enzymes and other kidney abnormalities

Therapeutic enzymes have also provided opportunities to explore novel therapeutic targets for CVD and CKD to discover new treatment regimens (Kumar et al., 2022). CVDs are the primary cause of death in CKD, suggesting that angiotensin inhibition caused a time-dependent increase in heart-ankle pulse wave velocity in non-diabetic CKD. In addition, it has been suggested that suppression of angiotensin led to an improvement in patient prognosis in non-diabetic CKD characterized by mild to moderate renal impairment (Mimura et al., 2008). Inhibition of some enzymes and activation of others are important regulatory strategies that can be used to slow or stop the progression of various kidney abnormalities (Granata et al., 2022). Extracellular superoxide dismutase (EC-SOD) is protective in CKD progression via reducing NOX activity and oxidative stress through β-catenin signaling in various kidney injuries (Tan et al., 2015).

Hemorrhagic shock is a common cause of mortality in significantly injured patients and could contribute to AKI, causing mitochondrial dysfunction by increasing ROS production. In the five-sixths nephrectomy (5/6NX) model, remnant nephrons show metabolic and hemodynamic responses to compensate for the renal mass loss, where the remnant nephrons undergo hypertrophy and hyperfunction. This condition contributes to ROS overproduction that induces mitochondrial dysfunction and CKD progression (Aparicio-Trejo et al., 2017). Treatment with resveratrol mitigates oxidative stress by decreasing mitochondrial ROS production and increasing SOD2 and CAT activity in hemorrhagic shock-induced Evans rats. Moreover, resveratrol treatment increased *SIRT1* and *PGC-1α* expression, restoring mitochondrial function (Wang et al., 2015). Administration of curcumin also suppressed oxidative stress by increasing *CAT*, *SOD*, *GR*, *GPX*, *GST*, and *Nrf-2* expression in nephrectomy-induced Wistar rats. Curcumin significantly protected mitochondrial function by decreasing *Drp1* and *Fis1* expression and increasing the activities of mitochondrial complexes I and V (Aparicio-Trejo et al., 2017).

It has been shown that edible phytochemicals and their main chemical components from *Camellia sinensis* (green tea), *Rubus idaeus* (raspberry), *Rubia cordifolia* (common madder), *Pistacia lentiscus* (mastic), *Petroselinum crispum* (parsley), *Punica granatum* (pomegranate), *Urtica dioica* (stinging nettle), *Solanum xanthocarpum* (yellow-fruit nightshade), *Dolichos biflorus* (horse gram), and *Nigella sativa* (black cumin) have received considerable interest in treating CKD (Nirumand et al., 2018; Hannan et al., 2021). In addition, other phytochemicals such as the antioxidant polyphenols catechin, epicatechin, epigallocatechin-3-gallate, diosmin, rutin, quercetin, hyperoside, and curcumin have been found to help prevent urolithiasis (Maditz et al., 2013). The main mechanisms through which these plants and their isolated phytonutrients help treat urolithiasis are their diuretic, antispasmodic, and antioxidant effects and their ability to stop crystals from forming, growing, and sticking together (Bland and Editor, 2017). Therefore, eating plants and polyphenols may help prevent and treat kidney stones. Further research is required to ensure that these compounds are safe and effective.

## Conclusion and future perspectives

Maintaining mitochondrial function has emerged as a potential strategy for ameliorating various human diseases, including kidney injury. This review highlighted how small molecule natural products could protect the kidneys from mitochondrial oxidative stress and mtDNA damage and improve mitochondrial biogenesis and dynamics in various kidney diseases, such as DN, HN, AKI, and CKD. Mitochondrial-targeted therapy is highly recommended to obtain maximum benefits, exploiting modern approaches such as nano-guided drug delivery. Further studies on the mechanism of action and therapeutic effects of small molecule natural products are required to enhance the viability of alternative therapeutic strategies in kidney diseases.
